# Sex-Biased Population Admixture Mediated Subsistence Strategy Transition of Heishuiguo People in Han Dynasty Hexi Corridor

**DOI:** 10.3389/fgene.2022.827277

**Published:** 2022-03-10

**Authors:** Jianxue Xiong, Panxin Du, Guoke Chen, Yichen Tao, Boyan Zhou, Yishi Yang, Hui Wang, Yao Yu, Xin Chang, Edward Allen, Chang Sun, Juanjuan Zhou, Yetao Zou, Yiran Xu, Hailiang Meng, Jingze Tan, Hui Li, Shaoqing Wen

**Affiliations:** ^1^ Ministry of Education Key Laboratory of Contemporary Anthropology, Department of Anthropology and Human Genetics, School of Life Sciences, Fudan University, Shanghai, China; ^2^ Institute of Cultural Relics and Archaeology in Gansu Province, Lanzhou, China; ^3^ Division of Biostatistics, Department of Population Health, School of Medicine, New York University, New York, NY, United States; ^4^ Institute of Archaeological Science, Fudan University, Shanghai, China; ^5^ Center for the Belt and Road Archaeology and Ancient Civilizations (BRAAC), Fudan University, Shanghai, China

**Keywords:** Hexi Corridor, subsistence strategy, sex-biased admixture, Y chromosome, mitogenome, ancient DNA

## Abstract

The Hexi Corridor was an important arena for culture exchange and human migration between ancient China and Central and Western Asia. During the Han Dynasty (202 BCE–220 CE), subsistence strategy along the corridor shifted from pastoralism to a mixed pastoralist-agriculturalist economy. Yet the drivers of this transition remain poorly understood. In this study, we analyze the Y-chromosome and mtDNA of 31 Han Dynasty individuals from the Heishuiguo site, located in the center of the Hexi Corridor. A high-resolution analysis of 485 Y-SNPs and mitogenomes was performed, with the Heishuiguo population classified into Early Han and Late Han groups. It is revealed that (1) when dissecting genetic lineages, the Yellow River Basin origin haplogroups (i.e., Oα-M117, Oβ-F46, Oγ-IMS-JST002611, and O2-P164+, M134-) reached relatively high frequencies for the paternal gene pools, while haplogroups of north East Asian origin (e.g., D4 and D5) dominated on the maternal side; (2) in interpopulation comparison using PCA and *Fst* heatmap, the Heishuiguo population shifted from Southern-Northern Han cline to Northern-Northwestern Han/Hui cline with time, indicating genetic admixture between Yellow River immigrants and natives. By comparison, in maternal mtDNA views, the Heishuiguo population was closely clustered with certain Mongolic-speaking and Northwestern Han populations and exhibited genetic continuity through the Han Dynasty, which suggests that Heishuiguo females originated from local or neighboring regions. Therefore, a sex-biased admixture pattern is observed in the Heishuiguo population. Additionally, genetic contour maps also reveal the same male-dominated migration from the East to Hexi Corridor during the Han Dynasty. This is also consistent with historical records, especially excavated bamboo slips. Combining historical records, archeological findings, stable isotope analysis, and paleoenvironmental studies, our uniparental genetic investigation on the Heishuiguo population reveals how male-dominated migration accompanied with lifestyle adjustments brought by these eastern groups may be the main factor affecting the subsistence strategy transition along the Han Dynasty Hexi Corridor.

## Introduction

Human history can be seen as a history of dealing with new challenges caused by changes of factors including resource distribution and social relationships. To overcome them, human beings have been using extrasomatic ways, including subsistence-, socio-, and ideo-technologies to create new niches for survival (Binford, 1962; Zhang 2021). Among the studies of adaptive changes, the evaluation of the factors leading to significant changes in subsistence strategy in human prehistory and history is a fascinating topic. Climate change, technological innovation, rapidly growing population, trans-continental cultural exchange, human migration, and geopolitics are clear potential candidates (Cohen, 1975; Bonsall et al., 2002; Gao et al., 2007; Pokharia et al., 2017; Cheung et al., 2019; Petraglia et al., 2020; Yang et al., 2020; Li et al., 2021). Climate change is well-studied and considered one of the most guiding factors of shifting subsistence strategies. A suitable climate will be conducive to the promotion of agricultural development, as rainy weather conditions facilitated the development of oasis agriculture in South-east Arabia around 5,100 cal BP (Preston, 2011), to take one example. On the other hand, an extraordinarily harsh climate can result in civilizational collapse. Humans are required to adopt different strategies, as well as adjust subsistence strategies, even migrate in search of better conditions, in order to cope with and adapt to such environmental changes (Polyak and Asmerom, 2001; Nunez et al., 2002; Haug et al., 2003; An et al., 2005; Preston et al., 2012; King et al., 2013; d’Alpoim Guedes et al., 2015; Jia et al., 2016; Pokharia et al., 2017).

The Hexi Corridor, located centrally on the eastern Silk Road, once played a crucial role in cultural exchange between east and west. The area was also a crossroads of agricultural and nomadic populations within China. Multiple lines of evidence showed that a significant shift in subsistence strategies along the Hexi Corridor occurred during the Han Dynasty (Ma et al., 2016; Yang et al., 2019a; Yang et al., 2020). Historical records from the *Shiji* (Records of the Grand Historian, 史记) to the *Hanshu* (Book of Han, 汉书) stated that the Hexi Corridor was occupied by nomadic pastoralists (Xiongnu, Yuezhi, and Wusun) before the Han Dynasty, a claim supported by excavated relics and faunal remains from Shajing culture (2,900–2,100 cal yr BP) and Shanma culture (2,700–2,100 cal yr BP) (Yang et al., 2019b) sites. The importance of domesticated pig declined precipitously during this same period (GPICRA, 2001), while sheep/goat, cattle, horse, and camel emerged as the major domesticated animals along the Hexi Corridor (GPICRA and SAMPU, 2011). A wealth of leather and woolen products had also been found at Shajing culture sites, suggesting how residents had initiated their own secondary products revolution with these domesticated pastoral animals (Sherratt, 1981). These were all indications suggesting that Hexi populations lived highly mobile nomadic lifestyles. During and after the Han Dynasty (202 BCE–220 CE), however, agriculture developed rapidly to become the subsistence strategy in this region. Numerous domesticated crop types had been found at archaeological sites, including barley, wheat, millet, highland barley, and pea (Sun and Liu, 2014), while advanced iron implements including iron plough, iron sickle, and iron spade had been excavated from numerous Han Dynasty sites (Yang, 2015). This strongly suggested that an advanced agricultural technology became widespread in the Hexi Corridor during this period of time. Remains of chicken, pigs, dogs, sheep/goat, cattle, and horse identified at the Heishuiguo Han Dynasty tombs should be placed in this context. Chicken would emerge as the most common domestic animal in this period, followed by pig (Li, 2021). This Heishuiguo population of domesticates resembled what we find with Central Plain farmers while being vastly different from that of nomads (the assemblage of domestic animals was sheep/goat, horse, and cattle) (Deng, 2015), who generally fed the camel and horse for long-distance migration, while pig and chicken may be more likely to appear in settled-peoples’ homes (Yang et al., 2019a). Finally, large numbers of painted murals depicting farming and animal-grazing had been found at Hexi Corridor sites, revealing this predominant Han-Jin Dynasties mixed economy (Zheng and Gao, 2019). We can be sure, therefore, that subsistence strategy in the Hexi Corridor shifted from nomadic style (pre-Han Dynasty) to a mixed style (during and post-Han Dynasty). The argument was also supported by stable isotope data (Li, 2021).

Located in an arid climatic transitional belt, the Hexi Corridor is extremely sensitive to changes in its environment. Previous studies have primarily attempted to explain shifting subsistence strategies through the mirror of environmental change (Zhou et al., 2016; Shi et al., 2018; Yang et al., 2019a; Yang et al., 2020). But we believe that the influence of population migration should not be overlooked, especially when considering the Hexi Corridor’s unique geographical position. Uniparentally inherited markers (Y chromosome or mitochondria) have been widely used in human population migration studies. The non-recombining portion of the Y chromosome (NRY) is strictly inherited paternally, while mtDNA is inherited maternally (Calafell and Larmuseau, 2017). MtDNA and Y chromosome therefore provide a matrilineal and patrilineal demographic history, respectively, revealing pictures of sex-specific processes in the past. Notably, genetic history revealed by Y chromosomes need not be identical to that by mtDNA. By using both mtDNA and Y chromosome inherited markers, sex-biased migration has been frequently found in studies of human populations and therefore might truly reflect the influence of social behaviors (Oota et al., 2001). So as to discuss the relationship between the change of subsistence strategies in the Hexi Corridor and population migrations during the Han Dynasty, here we analyze the Y-chromosome (including 485 Y-SNP markers) and mitochondrial genomes from 31 samples from the Heishuiguo site covering the whole Han Dynasty.

## Materials and Methods

### Materials

**FIGURE 1:**
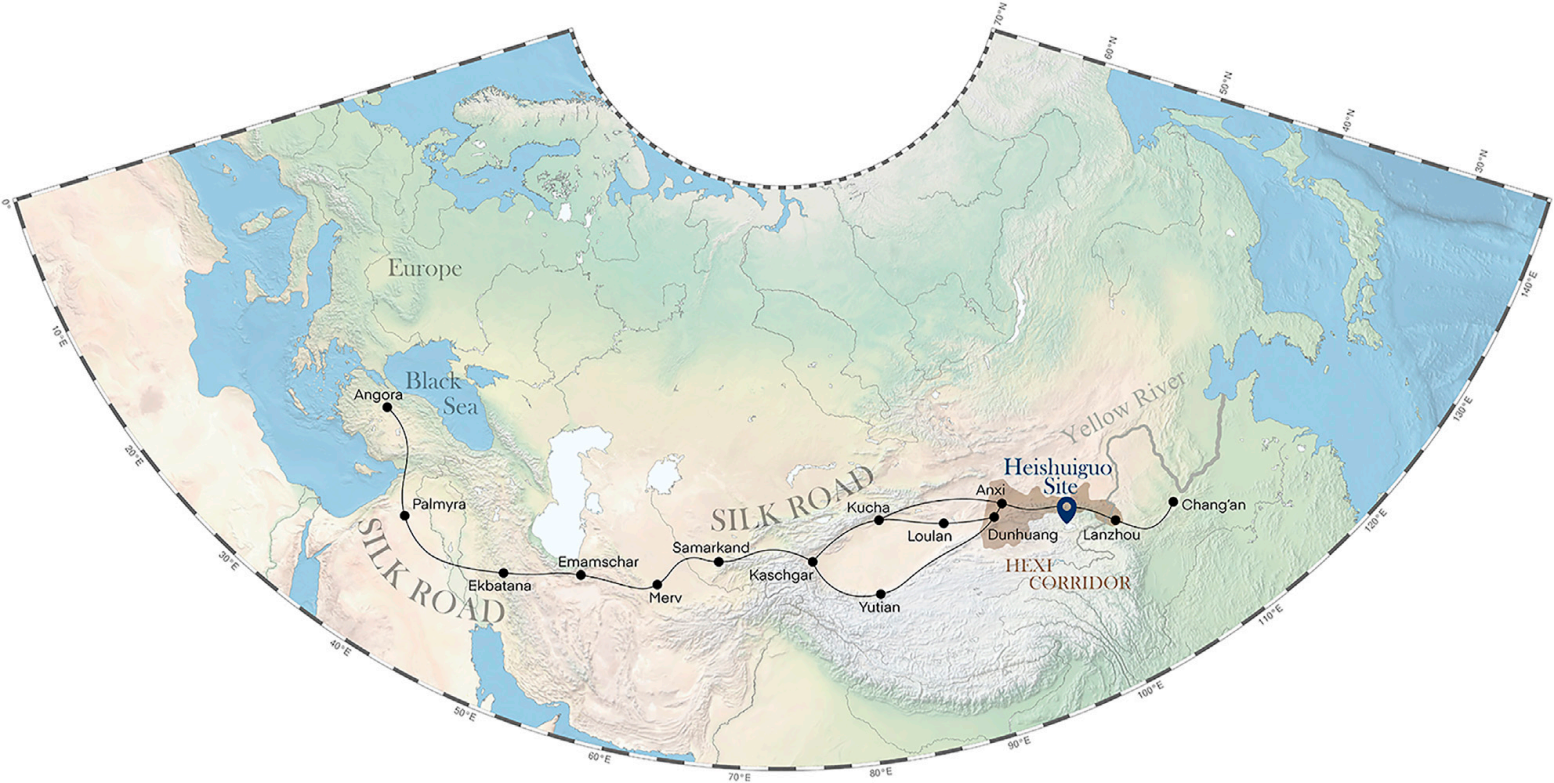
The geographical location of the Heishuiguo site and the Hexi Corridor.

The Heishuiguo site is located in Ganzhou county, Zhangye city, Gansu province, China (Figure 1). The site was excavated by the Institute of Cultural Relics and Archaeology of Gansu Province in 2018. Tomb distribution at Heishuiguo reveals a large cemetery consisting of family burial grounds and scattered burial groups (Chen et al., 2019). Based on ^14^C dating, tomb morphology and grave good assemblages (Li, 2021), burials at Heishuiguo have been divided into four phases: Phase 1, during the middle Western Han Dynasty (118–49 BCE); Phase 2, during the late Western Han Dynasty (48 BCE–6CE); Phase 3, from the Wangmang Xin Dynasty through the early Eastern Han Dynasty (7–67 CE); and Phase 4, from the middle to the late Eastern Han Dynasty (67–191 CE) (Zhang, 2017; Chen et al., 2019).

This study classified 31 individuals into two groups, the Early Han (Phases 1–2) and Late Han Dynasty (Phases 3–4), respectively. Heishuiguo individual sex was determined by pelvic (Klales et al., 2012) and skull morphology (Buikstra, 1994). Details are provided in Table 1 and Supplementary Table S1.

**TABLE 1:** Ancient individuals sampled in this study.

Sample ID	Archaeological ID	Periods	Skeletal element	5 C-T%	Sex (Genetic)	Contamination	Mt_Depth	Mt Haplogroup	Y-SNPs	Y Haplogroup
FA0211	M57 east	Early Han	Temporal bone	—	Male	—	0.109	—	480	O-F1759
G10105	M18 west	Early Han	Fibula	—	—	—	—	—	372	O-F325
FA0212	M54 east	Early Han	Temporal bone	12	Male	0.071	32.1589	—	480	O*-F996
EA1102	M4	Early Han	Temporal bone	10	Male	0.087	5.9048	B5a*	480	O-F8
EA1110	M23 west	Early Han	Clavicle	10	—	0.010	211.9229	B5b2a2*	480	O-F325
G10103	M15 south	Early Han	Fibula	5	—	0.089	20.7101	D4	413	C*-M217
FA0213	M30 east	Early Han	Temporal bone	10	—	0.061	30.1299	D4	480	O-F1736
EA1132	M25-2	Early Han	Tooth	11	Male	0.174	5.411	D4a6	478	N*-CTS439
EA1107	M19 west	Early Han	Temporal bone	10	Male	0.295	1.8525	D5a2a	477	O-F325
FA0205	M59	Early Han	Temporal bone	15	Male	0.019	54.9705	D5a2a1+@16172*	480	O*-F2924
FA0209	M57west	Early Han	Temporal bone	12	Female	0.066	17.3689	M11d	—	—
G30401	M33	Early Han	Tibia	14	Male	0.400	1.7359	M33c	445	O-F325
EA1101	M6①	Early Han	Temporal bone	14	Male	0.150	6.4597	R11a	454	O-F8
EA1130	M62 east	Late Han	Limb bone	—	—	—	0.0918	—	479	O*-F1365
G30705	M115 east	Late Han	Temporal bone	15	Male	0.018	59.0987	B4a1c3b	480	O-F1759
F11325	M84	Late Han	Fibula	17	—	0.009	89.4457	B5a2a1a	464	O-F1266
FA0210	M116 west	Late Han	Temporal bone	16	Male	0.023	41.4219	C4a1a2*	481	O-F1736
G10101	M13	Late Han	Limb bone	12	—	0.050	18.6234	D4a3b*	408	N-F710
EA0420	M90 west	Late Han	Tooth	19	—	0.002	361.4428	D4b2b*	480	C*-M217
EA1106	M98①	Late Han	Tooth	18	Male	0.001	730.8106	D4b2b*	480	N-F710
G30704	M113 north	Late Han	Temporal bone	16	Male	0.026	35.4385	D4j*	480	C-F5477
FA0206	M58	Late Han	Temporal bone	12	Male	0.018	30.8458	D5a2*	480	Q-1827
G40801	M79	Late Han	Limb bone	25	—	0.015	59.4633	D5b1b*	239	O-F141
FA0215	M38	Late Han	Temporal bone	14	Male	0.061	21.1436	D5c*	480	O-F8
G30703	M93	Late Han	Temporal bone	17	Male	0.016	60.0148	F1a1a*	480	O*-F1365
G30701	M5 west	Late Han	Temporal bone	11	Male	0.005	129.8333	G1c*	480	O-F4068
G30702	M9 north	Late Han	Temporal bone	13	Male	0.027	1.7359	G3	481	N-F710
FA0201	M80 north	Late Han	Temporal bone	13	Male	0.065	17.1204	M9a1a1b	480	O*-F46
EA1116	M92 east	Late Han	Limb bone	21	—	0.001	901.0238	N9a1*	251	O-F60
EA1104	M90 east	Late Han	Tooth	11	Male	0.069	7.898	R11b	480	C*-F3967
FA0214	M108 west	Late Han	Temporal bone	12	Male	0.033	29.8907	R11b*	481	O-F1759

### Methods

#### DNA Extraction

We extracted DNA from 31 samples using a dedicated aDNA facility at Fudan University, Shanghai, following the established precautions for working with ancient human DNA (Knapp et al., 2012; Sun et al., 2021). Negative extraction control samples (no sample powder used) were included, to monitor against contamination, as well as library negative controls (extract supplemented by water) in every batch of samples, which were processed and carried through the entire wet laboratory processing.

Prior to sampling, all samples were irradiated with UV-light for 30 min on each side and wiped with a 5% bleach solution. Teeth and other osseous materials were sandblasted to remove the outer surface before being ground to fine power with a mixer mill (Retsch, Germany). A dense section of temporal bones was cut around the cochlea by first removing the outer part, then grinding the clean inner part into fine powder. A total of 100 mg of bone powder was then used to extract DNA. Pre-lysis methods included the addition of a 1 ml extraction buffer, containing 0.5 M EDTA, 0.25 mg/ml Proteinase K (Merck, Germany), pH 8.0, after which samples were rotated for 1 hour at a temperature of 37°C. After centrifugation, the supernatant was discarded and 2.5 ml extraction buffer added, followed by overnight rotation at 37°C. We mixed 20 μl magnetic beads (Enlighten Biotech, China) with 12.5 ml binding buffer containing 5 M GuHCl, 40% Isopropanol, 25 mM sodium acetate, and 0.05% Tween-20 (Merck, Germany), (PH 5.2). We then transferred the supernatant (∼2.5 ml) to a binding buffer/bead mixture prior to the robotic extraction (Enlighten Biotech, China) procedure. At last, the DNA underwent elution through a 50 μl TET buffer (QIAGEN, Germany).

#### Library Preparation

We prepared double-stranded libraries in accordance with Meyer’s protocols (Meyer and Kircher, 2010), but with minor alterations. Libraries were amplified with indexing primers in two parallel polymerase chain reactions (PCR) using Q5 High-Fidelity DNA Polymerase (New England Biolabs, USA). Indexed products from the same library were pooled and purified using Agencourt AMPure XP beads (Beckman Coulter, Germany) and then eluted in 20 μl TET buffer. We quantitated the clean-up libraries using a Qubit 2.0 Fluorometer (Thermo Fisher, USA). We then sequenced the libraries on an Illumina HiSeq X10 instrument at the Annoroad Company (Beijing, China) using the 150-bp paired-end sequencing design.

#### Mitochondrial Capture and Sequencing

Target enrichment of the mitogenome was performed on each amplified library using a MyGenostics Human Mitochondria Capture Kit (MyGenostics Company, Beijing, China) as described by Sun et al. (2021), with hybridization and wash temperatures lowered to 60°C and 55°C to facilitate the enrichment of our short library molecules, in line with Dabney et al. (2013). A final post-enrichment amplification was performed for 15 cycles. The post-enrichment amplified product was then quantified via qPCR. Sequencing was performed using a Novaseq 6,000 platform at Mingma Technologies Company (Shanghai, China). Next, 150 bp paired-end reads were generated according to the manufacturer’s instructions.

#### Multiplex Polymerase Chain Reactions Targeted Amplification and Sequencing for Y Chromosome

**FIGURE 2:**
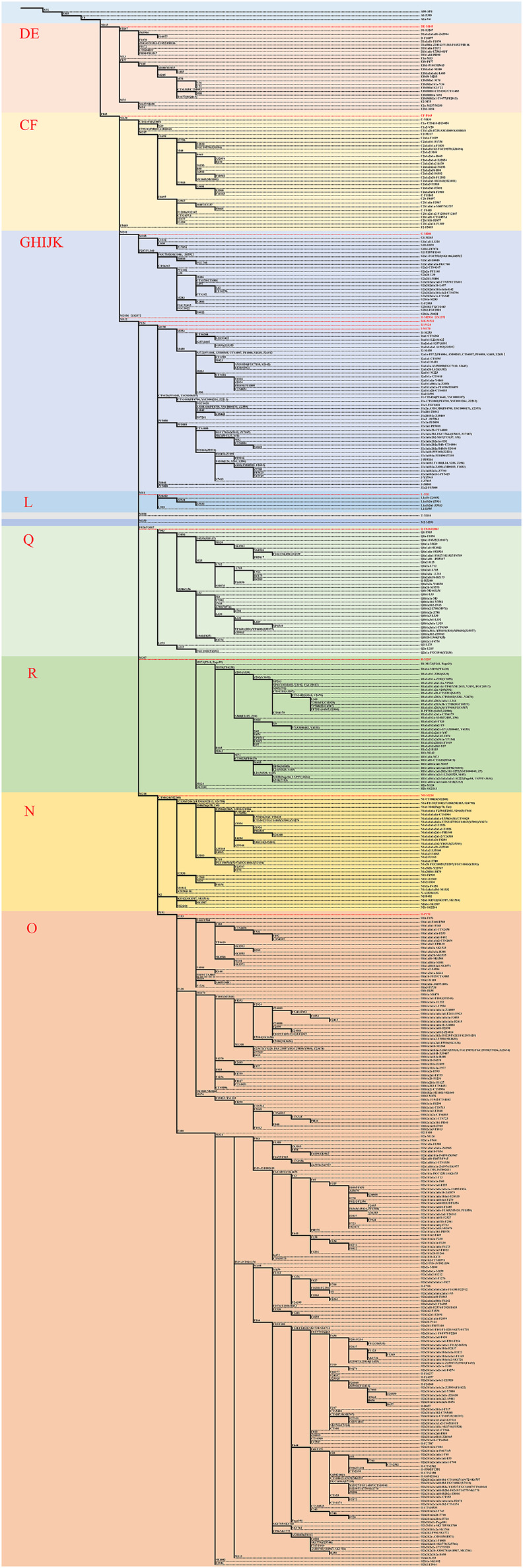
The phylogenetic tree of 485 Y-SNPs in this study.

Ancient DNA fragment lengths are generally skewed toward short fragments, the vast majority of which are typically shorter than 100 bp (Sawyer et al., 2015). Furthermore, in order to preliminary screen ancient samples quickly and inexpensively, we opted for multiplex PCR targeting enrichment with short amplicons based on the NGS (Next Generation Sequencing) platform. After amplification enrichment, a large number of samples could be detected and analyzed in parallel using the NGS platform. In view of the characteristics of highly degraded DNA involved in this study, we designed a more sensitive short amplifier primer system (Wen et al., 2019) and conducted tests on samples from the Heishuiguo site. The system comprises 485 Y-SNPs (Figure 2; Supplementary Tables S2, S3), covering common lineages in East Asia. Details can be found in the Supplementary Material. PCR amplification, sequencing, and data analysis can also be found in the Supplementary Materials.

#### Sequence Data Processing

For shotgun and mtDNA capture data, we clipped sequencing adapters and merged using sequences by ClipAndMerge v1.7.8 (Peltzer et al., 2016). Following this, we mapped merged reads to the human reference genome (hs37d5; GRCh37 with decoy sequences) using BWA v0.7.17 (Li and Durbin, 2010). PCR duplicates were removed using Dedup v0.12.3 (Peltzer et al., 2016). Using trimBam implemented in BamUtil v1.0.14 (https://github.com/statgen/bamUtil), we clipped four bases from both ends of each read to avoid an excess of remaining C->T and G->A transitions at the ends of the sequences.

#### Ancient DNA Authentication

The authenticity of the ancient genome sequence was mainly determined by the combination of two observations of the same specimen. Firstly, deep sequencing of the mitochondrial genome would show that the vast majority of the DNA fragments had come from a single individual. Second, the patterns of DNA degradation (Supplementary Figure S1), in particular nucleotide misincorporations resulting from deamination of cytosine residues at the ends of DNA fragments, would indicate that the mtDNA is ancient. We first checked DNA damage pattern and estimated the 5′ C>T and 3′ G>A misincorporation rate using mapDamage v 2.0.61 (Jónsson et al., 2013). We then made use of a Schmutzi program to test mitochondrial contamination rates for all individuals (Renaud et al., 2015).

#### Uniparental Haplogroup Assignment

For mtDNA, we employed a log2fasta program implemented in Schmutzi (Renaud et al., 2015) in order to call the mtDNA consensus sequences. Mutations that appeared when checked against rCRS were also re-checked in BAM (Binary Alignment Map) files through visual inspection with IGV software (Helga et al., 2013). Then, we used Haplogrep 2 (Weissensteiner et al., 2016) to assign the haplogroups.

Y chromosome haplogroups were examined by aligning a set of positions in the ISOGG (International Society of Genetic Genealogy, http://isogg.org/) and Y-full (https://www.yfull.com/tree/) databases, and analysis performed in the case of a base and mapping quality exceeding 30. Haplogroup determination was performed with the script Yleaf.py in Yleaf software (Ralf et al., 2018), which provides outputs for allele counts of ancestral and derived SNPs along a path of branches of the Y-chromosome tree. Finally, we re-checked the SNPs by visual inspection with IGV software (Helga et al., 2013).

### Statistical Analyses

Principal component analysis (PCA) was performed using FactoMineR package of R 3.6.3. Reference populations are listed in Supplementary Tables S4–S6. The pairwise genetic *Fst* was calculated using Arlequin 3.5 software. Heatmaps were also used to illustrate the clusters based on *Fst* by stats package of R 3.6.3. At last, to visualize the origins, we used PC1 values from PCA plot to generate contour maps with Surfer 12.0 software (Golden Software, https://www.goldensoftware.com/).

## Results

### Y-Chromosome and mtDNA Haplogroup Profile

**FIGURE 3:**
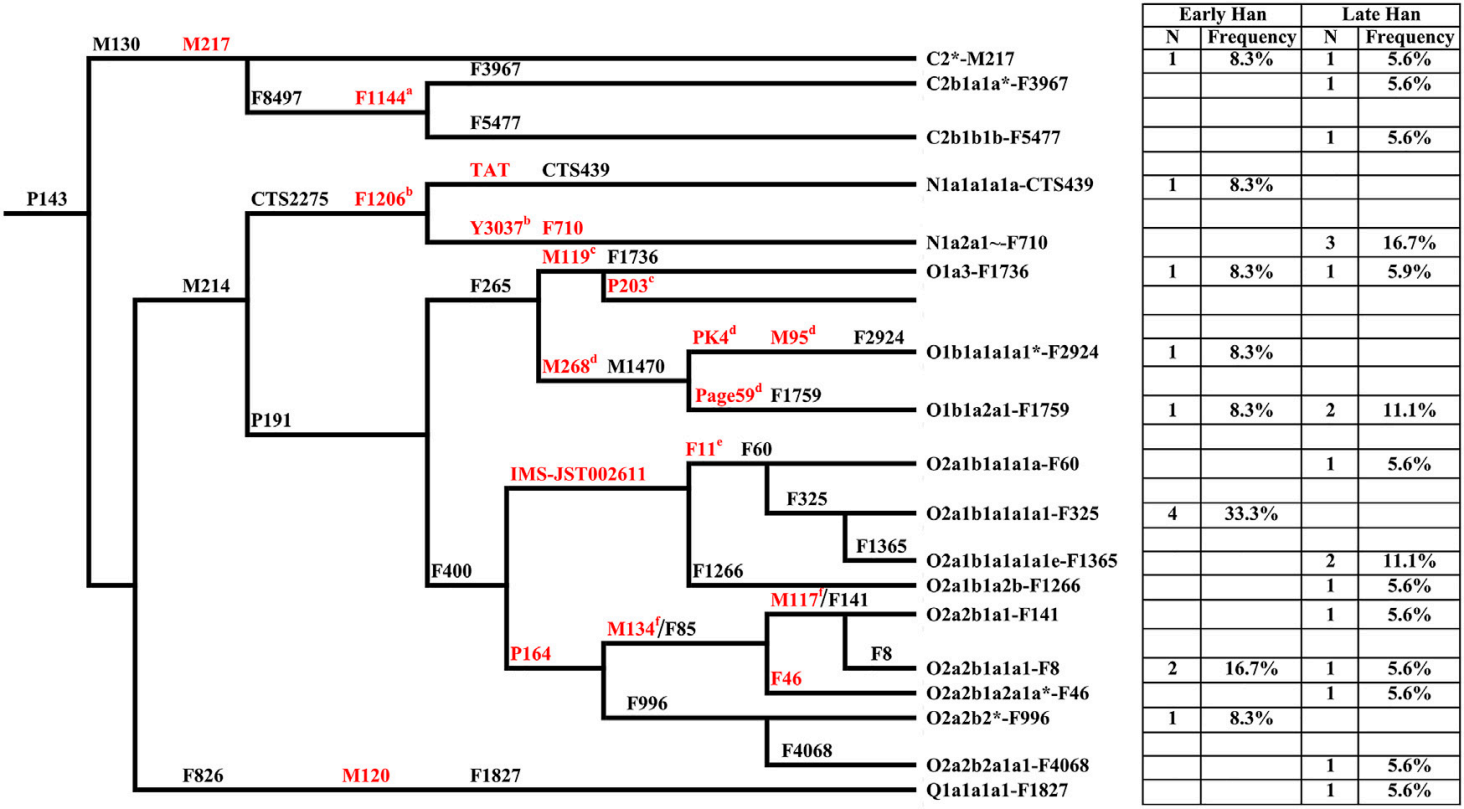
The phylogenetic relationship of Y-chromosome haplogroups in this study and their haplogroup-based frequencies in the sampled populations (Early Han, the early Han group; Late Han, the late Han group). Marker names are shown along the branches, and haplogroup names are shown to the right, based on ISOGG Y-DNA Haplogroup Tree 2019. Asterisks distinguish potentially paraphyletic undefined subgroups from recognized haplogroups. The markers in red are key Y-SNPs. a-f: These markers are not designed in Y-SNP panel but very common in Y phylogenetic trees.

Y chromosome haplogroups of 30 Heishuiguo males were determined according to the ISOGG’s Y-DNA Haplogroup Tree 2019 (Figure 3; Supplementary Table S1). Overall, the Heishuiguo population was comprised of the haplogroups Oγ-IMS-JST002611(26.7%), Oα-M117 (13.3%), C2-M217(13.3%), N-F1206 (13.3%), O1b1a2-Page59 (10%), O1a-M119+, P203- (6.7%), O2-P164+, M134- (6.7%), Oβ-F46 (3.3%), O1b1a1a-M95 (3.3%), and Q-M120 (3.3%). Haplogroups Oγ-IMS-JST002611 (26.7%), Oα-M117 (13.3%), and Oβ-F46(3.3%) represent three major founder paternal lineages (Yan et al., 2014; Wen et al., 2016) and encompass more than 40% of the present-day Han Chinese (estimated 16% for Oα, 11% for Oβ, and 14% for Oγ) (Yan et al., 2011). These three lineages have been considered to be derived from Neolithic farmers in Yellow River Basin (Yan et al., 2014). The three haplogroups are also the most frequent among the Heishuiguo population, representing 43.3% of the total, similar to modern Han populations. Haplogroup O2-P164+, M134-, may have expanded similarly with the above three haplogroups. Haplogroups C2-M217, N-F1206, and Q-M120 are common in Chinese, Altaic, Uralic, and Northern Eurasian Indo-European populations. The vast majority of haplogroup C in China belongs to C2-M217 (Zhong et al., 2010), constituting ∼10% of Han Chinese, as well as a great portion of Altaic-speaking populations, i.e., Mongol, Manchu, and Kazakh people. In the Heishuiguo population, the haplogroup C2-M217 can be further classified into C2*-M217 and C-F1144. The latter is a southern clade (Yan et al., 2014) and one of the six major paternal lineages of the Han Chinese (Wen et al., 2016; Wu et al., 2020). N-F1206 is called the northern clade of N haplogroup and widely distributed across Northern Eurasia (Hu et al., 2015). N-F1206 can be sub-divided into N-TAT and N-F710. The highest frequency of N-TAT is found in Altaic, Uralic, and Indo-European speaking populations (Ilumäe et al., 2016), such as Vilyuy Yakuts (91.525%), Evenks (50.877%), Buryats (41.441%), Udmurts (66.667%), Finns (53.846%), and Latvians (43.023%), while the N-F710 is considered as having migrated from Northeast Asia southward into the Yellow River region at no later than 2.7 kya (Ma et al., 2021). Haplogroup Q-M120 originated in South Siberia and expanded across northwestern China between 5–3 kya (Sun et al., 2019). The lineage was absorbed into ancient Huaxia (Han Chinese) populations before 2 kya and would eventually become one of the six founder lineages in modern Han populations (Wen et al., 2016; Sun et al., 2019). Finally, the O1b1a2-Page59, O1a-M119+, P203-, and O1b1a1a-M95 haplogroups, making up 20% of the Heishuiguo haplogroup proportions, are of minor southern East Asia origin (Karafet et al., 2010; Cai et al., 2011; Trejaut et al., 2014; Zhang et al., 2015; Luo et al., 2020). The paternal diversity of the Late Han Heishuiguo group exceeds that of the Early Han group, and features the addition of haplogroups N-F710, C-F1144, Oβ-F46, and Q-M120.

**FIGURE 4:**
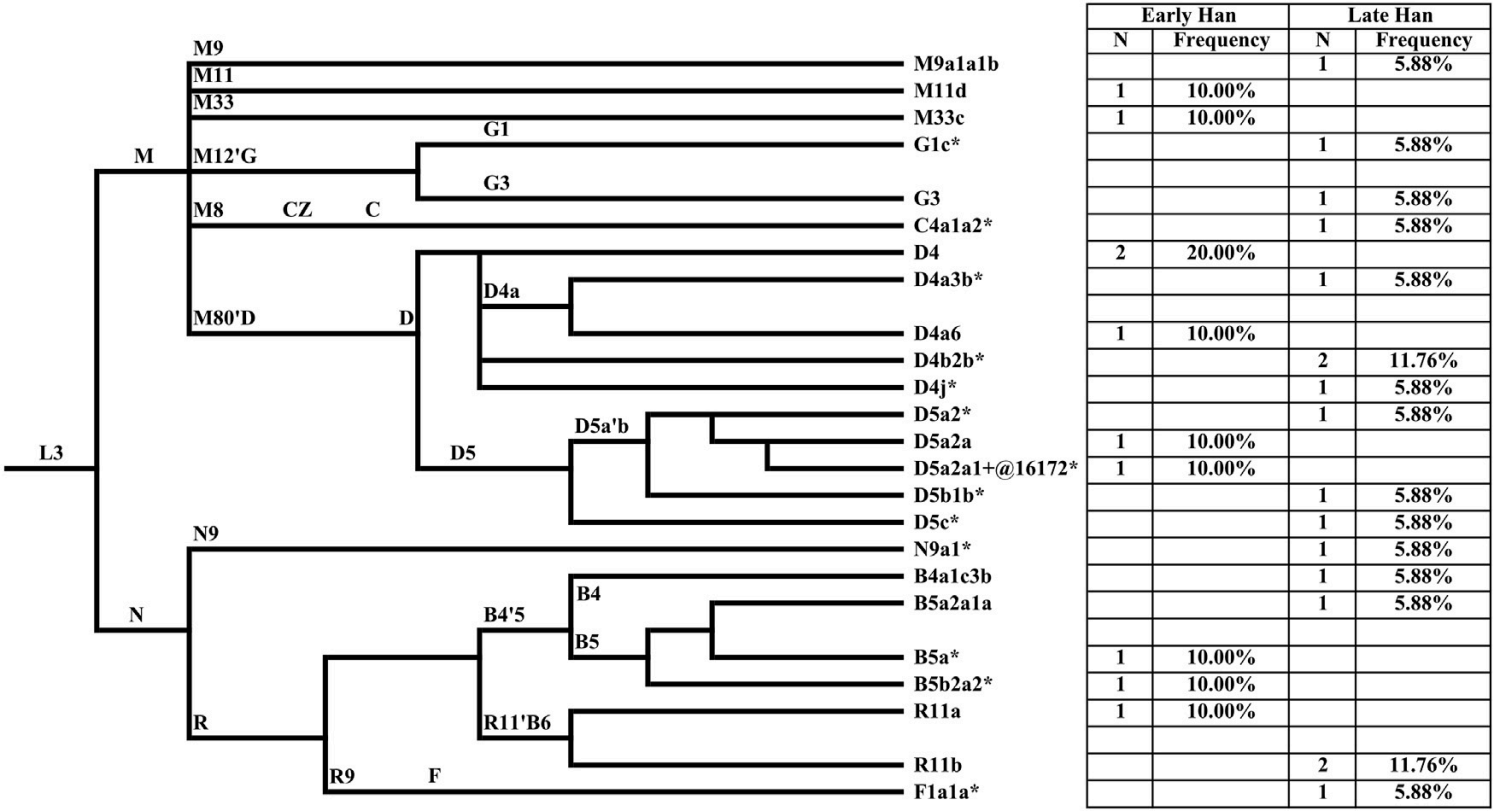
The phylogenetic relationship of mtDNA haplogroups surveyed in this study and their haplogroup-based frequencies in the sampled populations. Haplogroup names are shown to the right, according to the PhyloTree mtDNA tree Build 17.

MtDNA haplogroups of 27 Heishuiguo samples were determined based on PhyloTree mtDNA tree Build 17 (Figure 4; Supplementary Table S1). Different from the Y chromosome, the mtDNA gene pool is more heterogeneous. In the Heishuiguo population, these haplogroups consisted of D4 (25.93%), D5(18.52%), B5 (11.11%), R11(11.11%), B4 (3.7%), C4 (3.7%), F1(3.7%), G1(3.7%), G3 (3.7%), M11 (3.7%), M33 (3.7%), M9 (3.7%), and N9 (3.7%). Among these, haplogroups D4, D5, C4, G1, G3, M11, and M9 have origins in north East Asia. Haplogroup D4 occurs with the greatest frequency (25.93%) in the Heishuiguo population, and also retains a very high frequency in northern Asian (average 16.7%), central Asian (average 15.3%), and eastern Asian populations (average 22.5%) (Derenko et al., 2010). In this study, D4 could be further divided into sub-clades D4, D4a3b*, D4a6, D4b2b*, and D4j*. As for D5, this haplogroup spread across northern East Asia with moderate to low frequency and reaches its highest levels in Tubalar (25%) (Volodko et al., 2008), Beijing Han (15.38%) (Jin et al., 2009), Shannan Tibetan (15%) (Xu and Hu, 2015), Henan Han (11.4%) (Xu and Hu, 2015), Orochens (11.4%) (Kong et al., 2003), Koreans (10.4%) (Kong et al., 2003) from China, and Shandong Han (10%) (Yao et al., 2002). Here, D5 includes the sub-clades D5a2*, D5a2a, D5a2a1+@16172*, D5b1b*, and D5c*. These two dominant haplogroups, D4 and D5, with 44.44% of the Heishuiguo total, have a Northern Asian origin and distribution (Derenko et al., 2010). The remaining C4, G1, G3, M11, and M9 haplogroups are more common in northern East Asia than southern Eastern Asia. The B5, B4, and F1 haplogroups (occurring with 18.52% frequency at Heishuiguo) are relatively common in southern East Asia and may indicate gene influx from these regions. The haplogroup B5 retains high frequency in southern East Asia, especially in Tai-Kadai populations such as the Seak (69.23%) (Kutanan et al., 2017) and Kalueng (40%) (Kutanan et al., 2017) in Northeastern Thailand, Austro-Asiatic population like the Bru (45.83%) (Kutanan et al., 2017) from Northeastern Thailand, and Hmong-Mien population such as Hunan Yao (29.17%) (Wen et al., 2005) and Guizhou Han (10.19%) (Li et al., 2019). B5 is comprised of the haplogroups B5a*, B5a2a1a, and B5b2a2*. Likewise, the haplogroups F1 and B4 were common in southern East Asia and have southern Asian origins (Wen et al., 2005; Ko et al., 2014; Kutanan et al., 2017). Finally, the haplogroups R11, N9, and M33 occur sporadically in East Asian populations. We noted a similar degree of genetic diversity among both the Early Han and Late Han groups. In summary, the dominant mtDNA haplogroups at Heishuiguo, such as D4 and D5, are more frequent in northern East Asian populations and exhibit a northern Asian origin, while haplogroups B5, B4, and F1 might reflect gene flows from southern China.

### Population Comparisons

**FIGURE 5:**
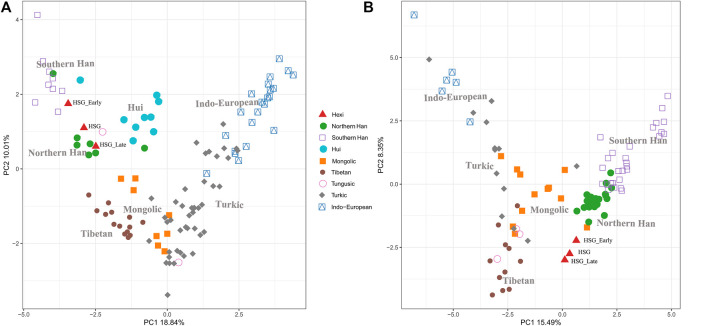
Principal component plots of Heishuiguo population and reference populations for Y-chromosome haplogroups **(A)** and mtDNA haplogroups **(B)**.

**FIGURE 6:**
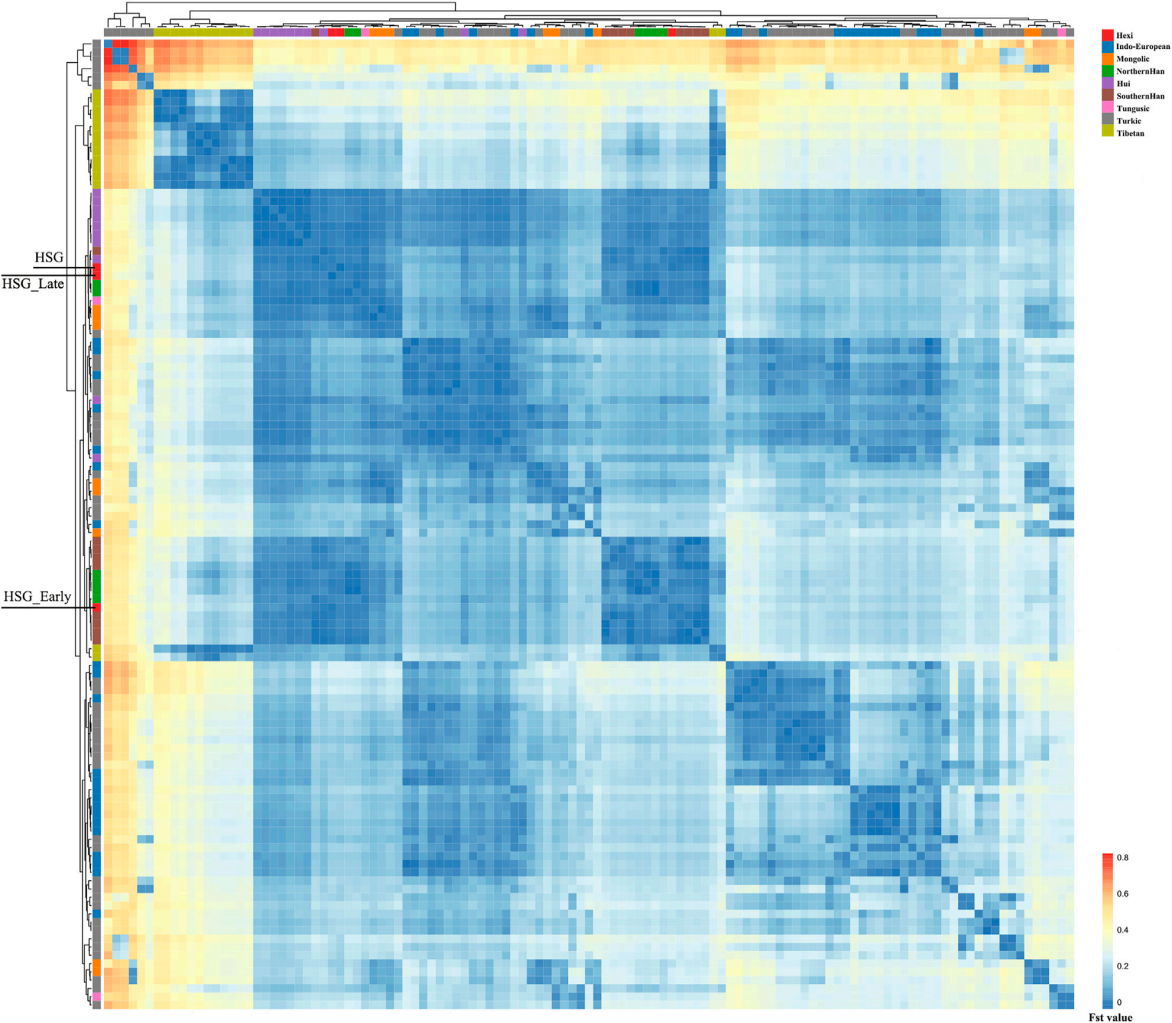
The genetic distance (*Fst*) heatmap plot of Heishuiguo population and reference populations for Y-chromosome.

**FIGURE 7:**
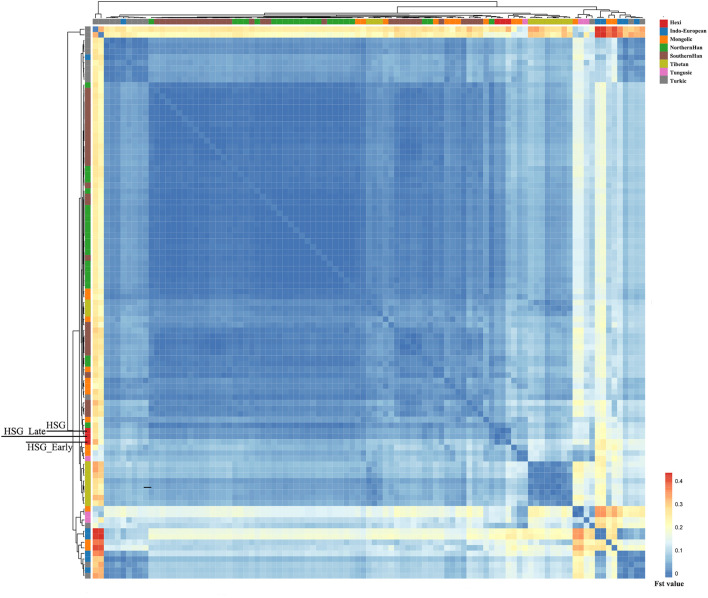
The genetic distance (*Fst*) heatmap plot of Heishuiguo population and reference populations for mtDNA.

In order to investigate the genetic relationships between Heishuiguo population and reference populations, we conducted a principal component analysis (PCA) based on haplogroup frequencies (Figure 5, Supplementary Figure S2; Supplementary Tables S4–S6). A genetic distance (*Fst*) heatmap was also visualized, to further explore population relationships (Figures 6, 7; Supplementary Figure S3). In this study, the Heishuiguo population was classified into three groups: Early Han, Late Han, and Overall group merging the former two groups. The PCA map (Figure 5) shows a division between the Early Han and Late Han groups according to PC1. Our Y-chromosome principal component plot (Figure 5A) reveals an overall Heishuiguo population is projected on to the Southern Han and Northern Han cline. More specifically, the Early Group appears to cluster around Southern Han populations, especially Fujian Han and Guangdong Han. According to previous studies, these two latter populations were descended from Northern China immigrations beginning in the Han Dynasty (202 BCE–220 CE) (Ge et al., 1997; Wen et al., 2004a). Our Early Heishuiguo population may therefore also reflect migration, in this case from the Central Plains to Hexi Corridor. The Late Group clusters closely with the Northern Han population in PCA plot and additionally clusters with Northern Han and Northwestern Hui in our heatmap (Figure 6), suggesting the genetic admixture with indigenous people over time. On the maternal side, the three Heishuiguo populations are tightly congregated (Figure 5B), mirroring genetic continuity from the Early Han to Late Han. Meanwhile, the Heishuiguo population maps out close to Mongolian-speaking (e.g. Buryat) and Northwestern Han (e.g., Ningxia Han) populations, which indicates an indigenous maternal origin.

In summary, an interpopulation comparison reveals that the Heishuiguo population shows close affinity with Han Chinese in terms of paternal structure and Mongolic and Northwestern Han populations in terms of maternal structure. This is a clear indication of sex-biased population admixture.

### The Origins of the Heishuiguo Population

**FIGURE 8:**
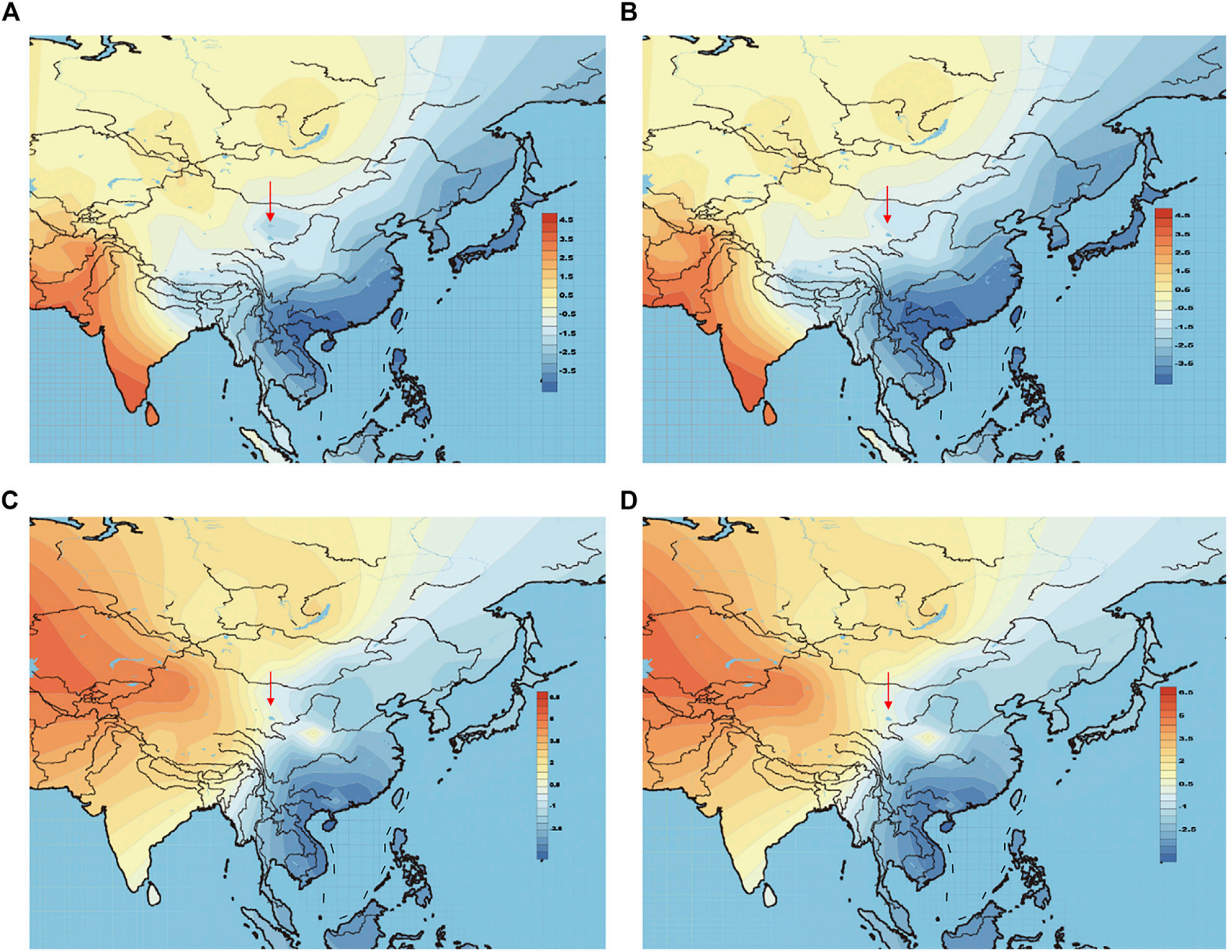
Genetic contour maps of Heishuiguo population and reference populations for Y-chromosome with early Han group **(A)**, Y-chromosome with late Han group **(B)**, mtDNA with early Han group **(C)**, mtDNA with late Han group **(D)**.

The above-mentioned PCA plot shows the eastern and western population cline according to PC1. We can make further use of PC1 values to generate genetic contour maps (Figure 8) and further visualize the possible origins of Heishuiguo population. On the paternal side, the contour map with early Heishuiguo group (Figure 8A) shows PC1 values gradually increasing from east-to-west across northwest China before dropping abruptly in the Hexi Corridor, a clue to the non-local and likely eastern origins of the population. Such a pattern does not repeat for the Late Han Heishuiguo group (Figure 8B). On the other hand, the maternal side paints a different picture (Figure 8C and Figure 8D), where we do not observe significant fluctuations between the Early Han and Late Han groups. This result points to the local roots of Heishuiguo females and their genetic continuity covering the whole Han Dynasty, coinciding with our PCA plot.

## Discussion

Multiple lines of evidence (i.e., historical records, archeological finds, and stable isotope analysis) have demonstrated how subsistence strategies along the Hexi Corridor shifted from a nomadic economy to mixed economy (i.e., pastoralism and farming) from the Han Dynasty (Sun and Liu, 2014; Li 2021). Prior research has focused on exploring the relationship between climate change and subsistence strategy transition in this region during the Han, suggesting a cold and arid climate had dried up considerable stretches of river and impoverished fertile lands prior to the Han Dynasty, with resulting prevalent nomadism. A warmer and wetter climate from the Han onwards promoted the thriving of agriculture (Yang et al., 2020). Up till now, it has seemed farfetched to link this transition to climate factor simply.

On basis of Y chromosome and mtDNA profiles of Heishuiguo population, we have located a Heishuiguo patrilineal population consisting of Yellow River Basin origin haplogroups (i.e., Oα-M117, Oβ-F46, Oγ-IMS-JST002611, and O2-P164+, M134-) at an overall rate of over 50%, along with southern East Asian origin haplogroups (O1a and O1b) at ∼20% and northern East Asian origin haplogroups (C2-M217, N-F1206, and Q-M120) at ∼30%. The matrilineal Heishuiguo population consisted of northern East Asian haplogroups (e.g., haplogroups D4, D5, and C4), which accounted for ∼62.95%, alongside southern East Asian haplogroups (such as B5, B4, and F1) at 18.52%. By way of interpopulation comparisons (PCA and *Fst* heatmap) with reference populations (e.g., Southern Han, Northern Han, Hui, Mongolic, and Tibetan), we have been able to show closer paternal genetic affinity with Northern Han and Hui populations among the groups at Heishuiguo. Via PCA, we observed genetic structure changes from Southern-Northern Han cline to Northern-Northwestern Han/Hui cline with time (Figure 5A), indicating genetic admixture between Yellow River immigrants and natives. Historical records and archaeological finds add further credence to our results. According to historical documents (Qi, 1983; Si, 2002; Ban, 2008; Bamboo Slips Museum, 2013), the Han Dynasty government migrated population on a large scale from about 20 counties in Yellow River Basin to four counties (i.e. Zhangye 张掖郡, Jiuquan 酒泉郡, Wuwei 武威郡, and Dunhuang 敦煌郡) in Hexi to strengthen administration and control over this region. Bamboo Slips (简牍) provide more detail and even give us the possible source of these male immigrants in 21 counties from the Middle and Lower Yellow River (Hongnong 弘农郡, He’nei 河内郡, Langya琅邪郡, Changyi 昌邑国, Pinggan State平干国, Dahe 大河郡, Chenliu 陈留郡, Runan 汝南郡, Julu 巨鹿郡, Yingchuan 颍川郡, Shangdang 上党郡, Henan 河南郡, Jiyin 济阴郡, Nanyang 南阳郡, Hedong 河东郡, Zhao State赵国, Dong 东郡, Liang State梁国, Zhangye张掖郡, Huaiyang 淮阳郡, and Wei 魏郡) (Liu, 2012; Li, 2018, Figure 9). Our view from the maternal mtDNA (Figure 5B), however, shows the Heishuiguo population closely clustered with certain Mongolic and Northwestern Han populations and exhibiting a genetic continuity covering the whole Han Dynasty, suggesting a possible local origin for Heishuiguo females. This is in accordance with historical records (Liu 2012), where major migration events were often male-dominated migratory and frequently involved migration for garrison building, political migration, and migration of minority groups. Young males were usually the ones building garrisons, and most couldn’t bring their families outside of small local garrisons. Political migration involved political prisoners, ordinary crimes, and victims of natural calamities. Migration of minority groups targeted rebels from border areas such as Di and Qiang peoples in the Upper Yellow River Basin. Among them, the military migration was in the majority. This is likely why a sex-biased admixture pattern can be clearly observed in the Heishuiguo population. Such sex-biased admixture patterns have also been observed in population expansion of Han and Tibeto-Burman-speaking (Wen et al., 2004a; Wen et al., 2004b) people. We plotted genetic contour maps to visualize the possible origins of Heishuiguo population. As shown in Figure 8, we can easily observe that the primary eastern East Asian origin of male ancestry and native origins of female ancestry.

**FIGURE 9:**
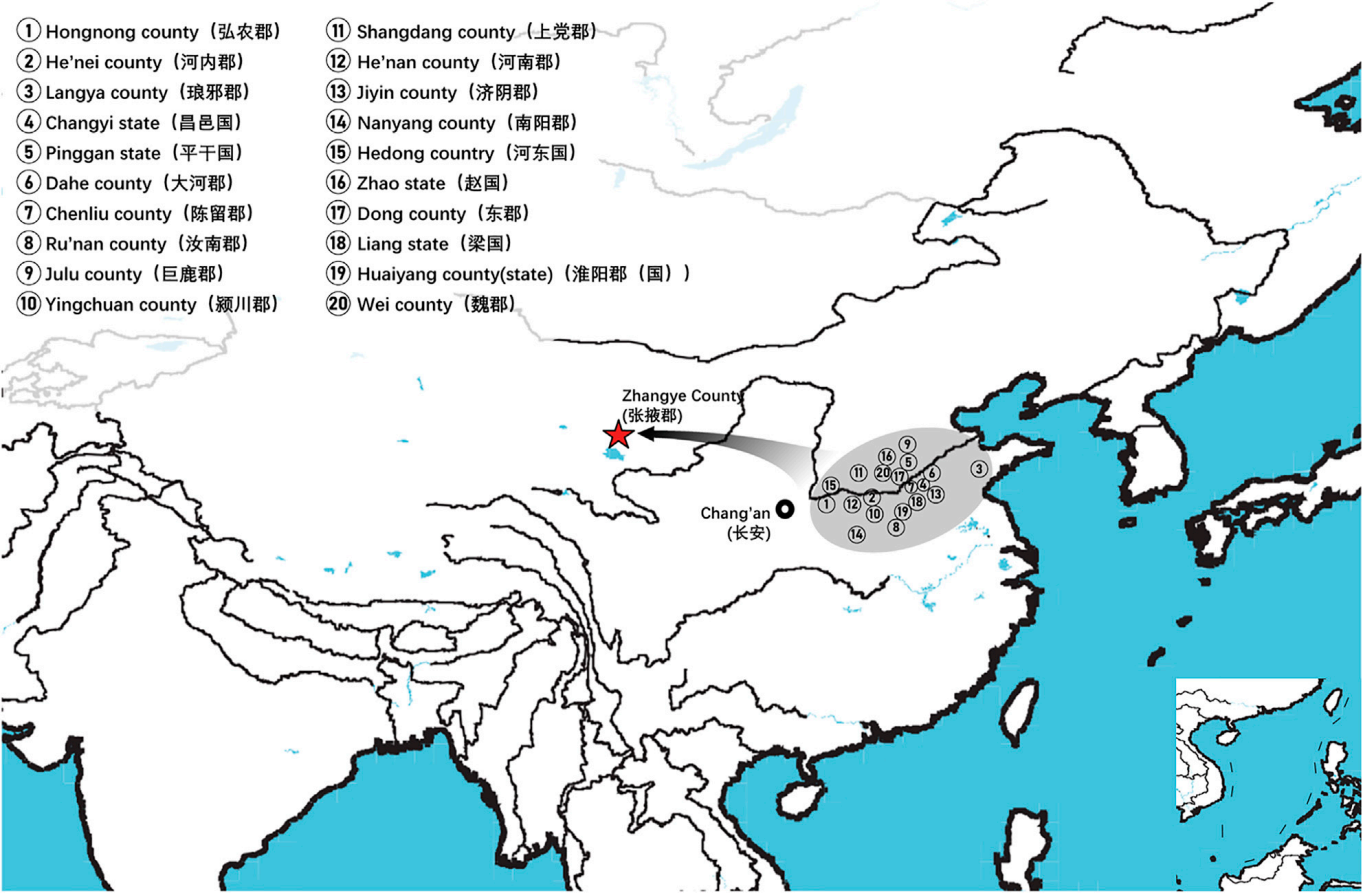
Immigrants from 20 counties in middle and lower Yellow River to Hexi Corridor according to the contents of unearthed bamboo slips.

In this study, by means of a uniparental genetic analysis, we have observed a male-dominated admixture event occurring during this period, one additionally supported by historical records and archaeological findings. That shifting subsistence strategy along the Hexi Corridor residents that kept pace with human migration cannot be coincidental. Mass migration of individuals and transplantation of subsistence lifestyles would have impacted the former subsistence strategy in Hexi Corridor during the Han Dynasty. This study provides new insights and possibilities into how population admixture serves as a key factor in changes of subsistence strategy.

## Data Availability

The datasets presented in this study can be found in online repositories. The names of the repository/repositories and accession number(s) can be found in the article/Supplementary Material.

## Supplementary Material

Supplementary Figure S1 Ancient DNA damage patterns. Nucleotide misincorporation patterns cause by C deamination in ancient DNA sequences.

Supplementary Figure S2 Principal component plot of Heishuiguo population and published ancient populations for mtDNA haplogroups.

Supplementary Figure S3 The genetic distance (*Fst*) heatmap plot of Heishuiguo population and published ancient populations for mtDNA.
