# Supplementary figures and images for: Sex-Biased Population Admixture Mediated Subsistence Strategy Transition of Heishuiguo People in Han Dynasty Hexi Corridor
Source: Front Genet. 2022 Mar 10;13:827277. doi: 10.3389/fgene.2022.827277 (PMC8960071; doi:10.3389/fgene.2022.827277)

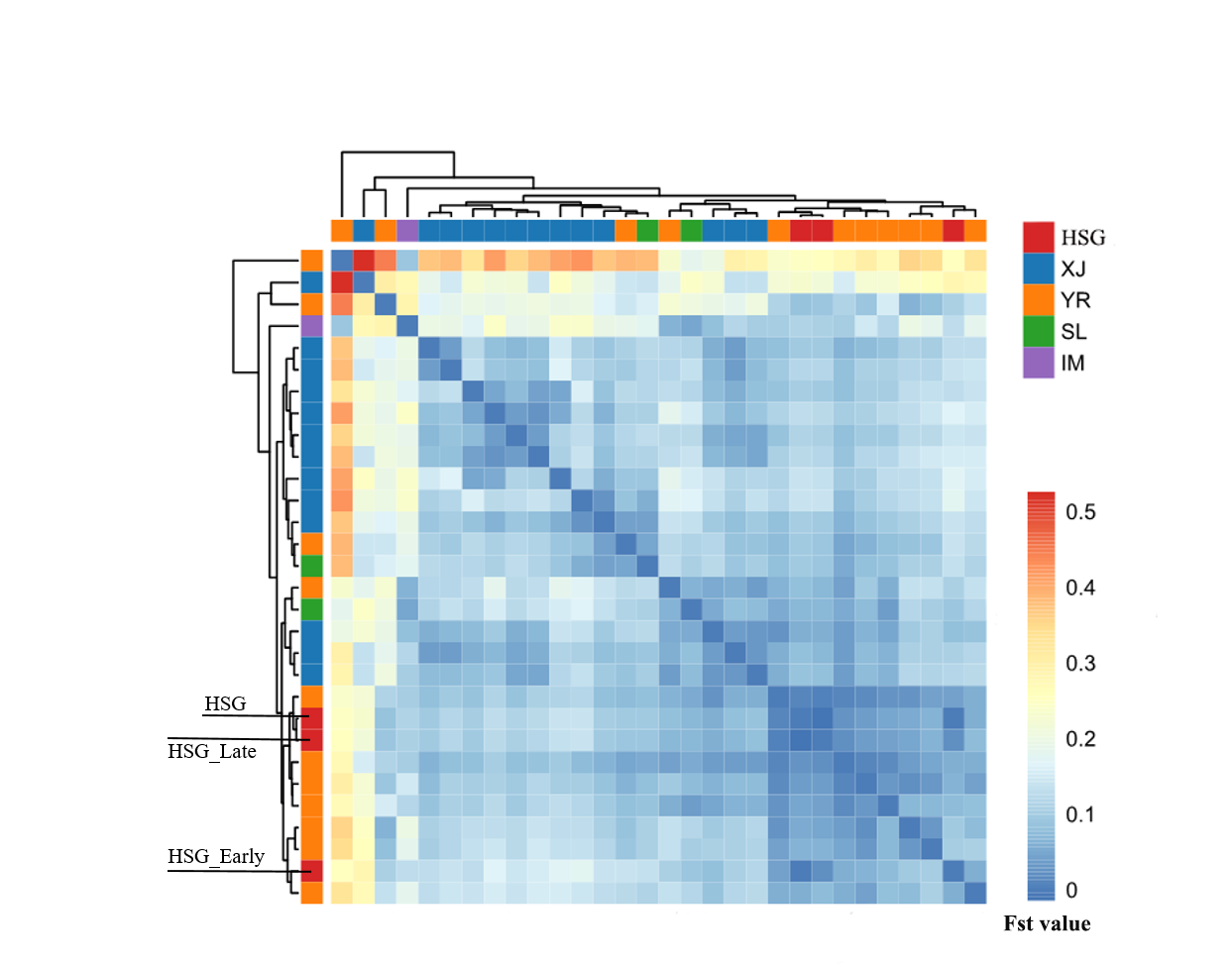

Supplement: Supplementary file 1 [file Image3.TIF]

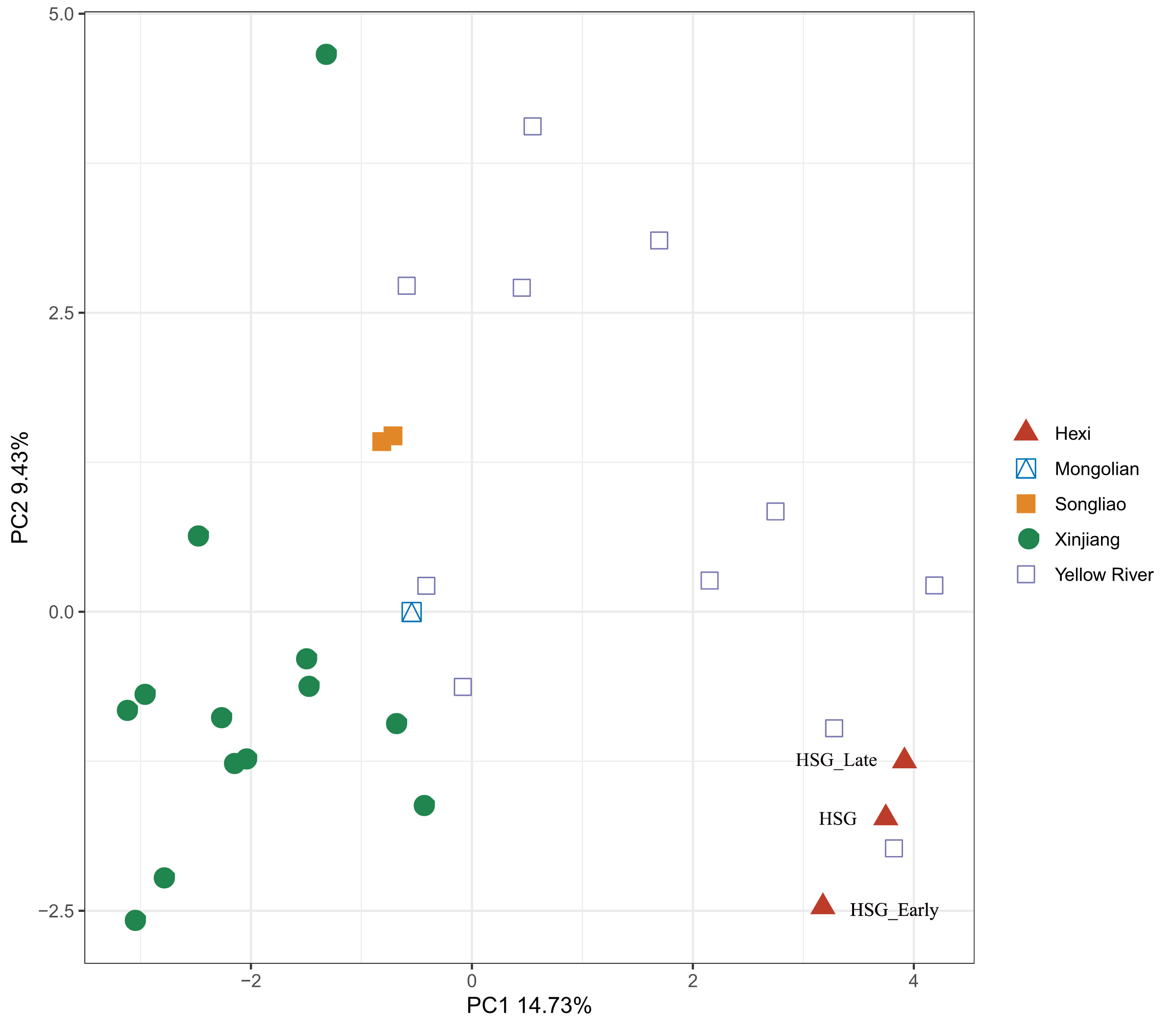

Supplement: Supplementary file 2 [file Image2.TIF]

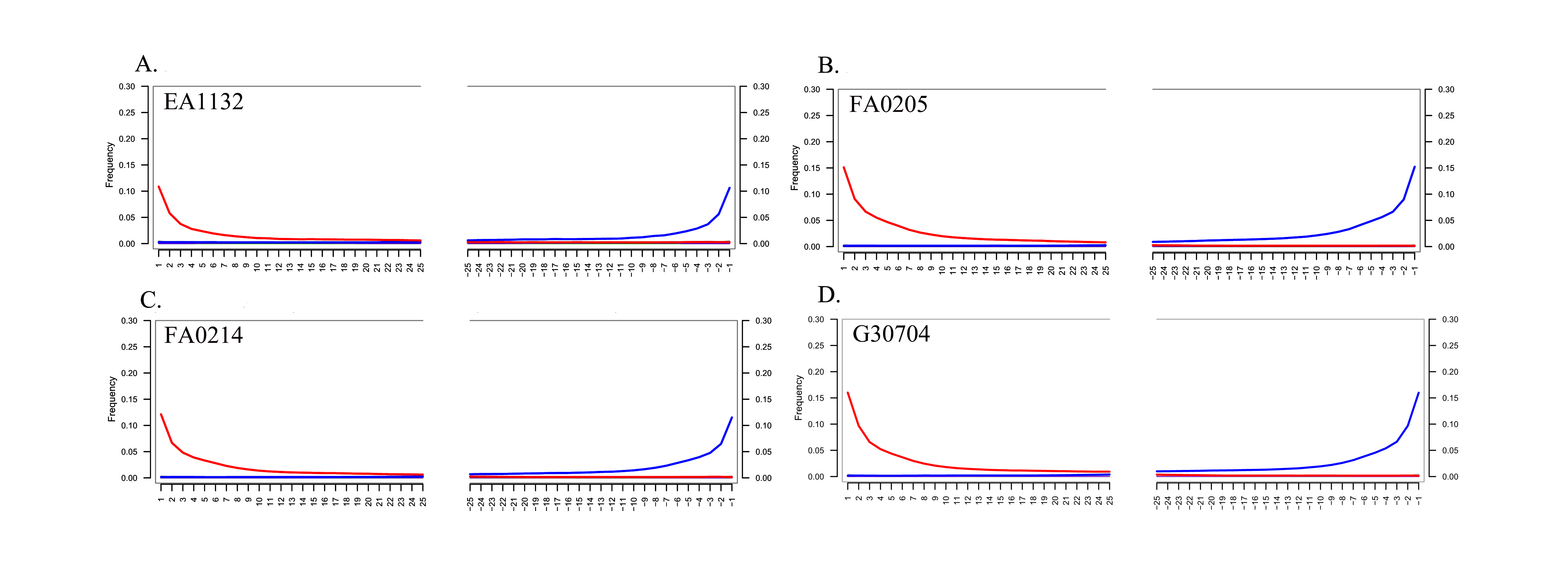

Supplement: Supplementary file 3 [file Image1.TIF]
