# Supplementary material for: Sex-Biased Population Admixture Mediated Subsistence Strategy Transition of Heishuiguo People in Han Dynasty Hexi Corridor
Source: Front Genet. 2022 Mar 10;13:827277. doi: 10.3389/fgene.2022.827277 (PMC8960071; doi:10.3389/fgene.2022.827277)
Supplement: Supplementary file 4 [file DataSheet2.docx]

1. **Panel Design**

In the preliminary experiments, we found that the DNA from archeological remains was highly degraded, and the length of DNA fragments mostly concentrated in the range of 70-150 bp. Therefore, we chose multiplex PCR targeting capture based on the NGS platform to ensure the uniformity of amplification. After amplification, a large number of samples can be detected and analyzed in parallel using NGS platform. In view of the characteristics of highly degraded DNA involved in this study, we designed a more sensitive short amplifier primer system.

The panel comprises 485 Y-SNPs, which covers the common lineages in East Asia. The details are as follows.

**485 Y-SNPs**：M176, Z17874, F708, Z35983, F4535(Z19137), F710, CTS53, CTS66, F8465, P305, F717, B482, SK1514, B470, M130, F723, F725, M406, M407, F728, PH40, F8, Y26395, F8497, F3830, M324, P201, A2828413G, F13, F743, F26868, F700, Z6616, PH90, M346, M343, F748, Z2054, Z7299, A9461, PF3146, Z8022, F3737, PH116, C6651181T, A6695160G, CTS342, F810, F813(M6539), F819, Z35160, CTS426(PF4641,YSC0000307), CTS439, B433, F826, K1512(SK1517, SK1514), F5477, F827, F830, V36, FGC39579(Z31694), V20, L940(F835), V4, Z26015, L712, CTS579, V22, F46(Y15), L830, F4065, Z7700, CTS595, AF6, B457, F854, F48, F856, CTS616, CTS623(PF6419), F866, AM01856(F871), G6942166A, TS677(PF2015), SK1512, Z20492, SK1770, CTS713, Z43879, F55, CTS723, F4068, SK1507, BZ175, F903, F906, F6193, CTS901, Z24014, F915, F60, M522, F919, F923, S218(Z253), CTS1102, Y7582, CTS1174, CTS1211(S3357), F940, CTS1303, Z43967, C7383403T, CTS1451, F964, CTS1483, F977, F979, IMS-JST002611, F993, F996, F85, B388, Z34163, F1019, F1022, M1932,F16077, A9472, F4084, PF5008, Y16313(Z35310), YP1541, C7765756A, SK1726, B456, FGC16863(Y7110), F100, Z8806, Y26383, F1070, FGC10851(Y5297), F16381, FGC6031, F1095, F1096, PF5088, B80, F5489, L56, K473, F1123, Z35348, SK1927, F1127, PF5160(Z2221), Z43904, F4759, SK1772, F134, M5415, F5481, F1212, F138, F5504(SK1630), F141, F1232, F1236, L1324, SK1676, F1252, B469, F1262, B115, F1266, Z396, Z780(M971), YP968(FGC4517), F14326, F1273, F1276, F4774, F1290, F153, SK1778(Z25746), F1313, F5506(SK1636), L22(S142), F27531, FGC7535（SK1106，Z6552）, Z25907, F1365(M5420, PF1558), F1369, Z781, PF5090, Z32854, F1388, F1389, F1391, M405, F168, F27587, L485, Y20935, PF6896, Z1926, Y1274, Z5933, B450, FGC10863(Y3191), F1536, F201, Y13527, Z18440, B170, F4156, Z25942, F6592, Z26030, F11165, Y17949, JN15, P164, F1675, Z400(AM00103, F1683), Z24089, F238, Z25930(F14422), CTS1936, F1699, P177, PH975, L596, P143, CTS2190, F1736, AMM050(FGC7110, S2645), F4168(L24, S286, Z396), F1756, CTS2457, CTS2458, F254, F1759, CTS2562, FGC3022, P37.2(PF4004, AM00519, CTS4097, PF4008, S2669, Z2651), F1803(M1348), Z6046, F1813, CTS3068(PF4700, YSC0001264, Z2213), CTS3085, F1827, M333, M170, M180, N5, F270, F1852, Z43976, L215, CTS3402(S3361, V2670), M184, M222(Page84, USP9Y+3636), FGC14165(Y5001), CTS3427, M46(Page70, Tat), F1863M237, M188, AM01310(PF4708, YSC0000173, Z2359), M250, M253, M173(P241, Page29), M201, Page101, M198(PF6238), L713, M283, F1918, FGC17664(Y5035, Z17187), Z2651, F1942, P191, M217, CTS4179, M215, M214, Y24368, FGC12511, M207, Z282(V3055), L30, CTS4367, L21(M529, S145), Z43977, CTS4585, Y920, CTS4693, CTS4800, YP417(M12415, V3192, FGC20517), YP1691(B30), CTS4960, F310, SK1734, M1368, F317, SK1555, PF7521(S4567,Z2908), Y2910(FGC10329), F325, L332, F4199, PF6899, SK1675, CTS5488, F2130(F2042), F2137, SK1568, F2172, Z5931, PF5425, M438, L42, F4212, Z23673(Y9320, FGC29897), F2268, CTS6364, SK1661(SK1601), F5484, AM01781(S18967, SK1786), F12086, F2356, SK1746(F5524), CTS6796, CTS6803, CTS6804, F12147, SK1660, Z24088, CTS6904, F2411, F2415, F4229, M353, F400, S19575, S272(YSC0000049, Z7), SK1755, F3960, L497, F2489, F2491, F2527, S205(Z92), F4370, F3967, SK1922, FGC1846(Y2136), F2569, Z43965, PH3317, DF96(S1809), FGC32413, F2576, PH3340, F2584(F2065, S20163), L330, M2936(Z4237), F4249, F438, Y57, PF5190, F444, F446, F449, F4251, CTS8428, F2685, Y47, Y4166, SK1926, Y874, Z7415, CTS8624(M2240), S337（Z60）, Y16850, P180, F492, F12502, L621(S392), F22912, FGC29898(Y9036, Z23674), F2859, F2868, SK1533, F2883, L366, F2890, F4274, SK1802, F2924, P124, F2928, F2930, M423, M3, BZ380, F2941, YP4610, F2944, L275, F4284, CTS9996, F533, YP1692(Z35977), Y9, Z25928, Y7080, F3067, F3053, CTS10427, Y7(AM00482, V4155), CTS10535, CTS10573, S340(F3105, Z94), F560, Z1842, SK2264, Z4056, SK2163, F3163, L550(S431), Z39487, F3172, Z25910(F1455), YP4549, Z1936, F24357, YP263, L53, M145, M223, M101, SK1757, M11, L595, M33, F3247, M48, M159, SK1731, M124, L329, M25, M54, M67(PF5137, S51), M73, M75, M81, M78, L715, M92, M110, M120, Z283/(S339), P287, S1953（Z2535）, SK1760, K644, PH5117, B418, M1470, FGC766, Y16779, SK1764, PF7261, CTS10738(M1707), Y23787, M285, CTS10841, Y3640, F3361(M2113, S24798), PF5216, SK1571, CTS11043

1. **PCR Amplification and Sequencing**
   1. **Multiplex PCR**

Setting up the multiplex PCR should be carried out in a specialized ancient DNA laboratory. All surfaces and equipment should be cleaned with bleach and ethanol to avoid contamination. Always include one PCR blank per eight to ten samples.

| Reagent | Volume per reaction (μL**)** |
| --- | --- |
| Nuclease-free water | Add up to 50 μL |
| Primer Master Mix | 6 μL |
| gDNA(1-200 ng) | x μL |
| 3 x EnzymeHT | 17 μL |

After PCR setup, run the multiplex PCR in a thermocycler: initial denaturation at 98℃ for 3 min, followed by 32 cycles of denaturation for 20 s at 98℃, annealing & elongation for 6 min at 64℃,. The protocol ends with a final extension at 72℃ for 2 min.

- 1. **Purification of PCR products**

Purify the reactions using the Agencourt AMPure XP Reagent beads according to manufacturer’s instructions. At last, using a pipette, remove residual traces of ethanol. Let the beads air-dry for 8-10 min at room temperature without caps. The DNA attached in beads in this step will be the template for indexing PCR.

- 1. **Indexing PCR**

Prepare the master mix by adding all reagents except the barcoded indexing primers. Then add the master mix into PCR tubes from the purification step. Mix carefully by pipetting. At last, add the indexing primer.

| Reagent | Volume per reaction(μL) |
| --- | --- |
| Nuclease-free water | 18 μL |
| Primer_LF(10 μM**)** | 1 μL |
| Indexing Primer(10 μM) | 1 μL |
| 3 x EnzymeHT | 10 μL |

After PCR setup, run the indexing PCR in a thermocycler: initial denaturation at 98℃ for 2 min, followed by 8 cycles of denaturation for 15 s at 98℃, annealing for 30 s at 55℃, elongation for 30 s at72℃. The protocol ends with a final extension at 72℃ for 2 min.

- 1. **Purification of PCR products**

Purify the reactions using the beads according to manufacturer’s instructions. Elute and store DNA in 20 μL HPLC grade water.

- 1. **Quantification**

Quantify the purified amplicon libraries with the Qubit® dsDNA HS Assay Kits according to manufacturer’s instructions. Then Pool all sample libraries using an equal amount of DNA.

- 1. **Sequencing**

Measure the DNA concentration of the pooled library on a Qubit fluorometer according to manufacturer’s instructions, and dilute to the required concentration. Sequence purified and pooled amplicon libraries on Novaseq 6000 sequencers according to manufacturer’s instructions.

1. **Data Analysis**
   1. Novaseq 6000 platform was used for double-ended sequencing to obtain sequence data of 150-bp paired-end reads (fastq).
   2. We clipped the sequencing adapters by cutadapt software.
   3. After adapter removing, reads were aligned to hg19 by bwa (parameter: aln) (version 1.7) to generate BAM (Binary Alignment/Map) files.
   4. For the new BAM file, we used BAMClipper software (Au C H et al. 2017) to get the final BAM file by removing the primer sequence from the BAM file according to the primer’s physical location.
   5. We used samtools (version 1.8) and bcftools (version 1.8) to identify mutation sites (vcf).
   6. We checked the mutations by using IGV (2.5.0).
